# A case series on common cold to severe bronchiolitis and pneumonia in children following human metapneumovirus infection in Sri Lanka

**DOI:** 10.1186/s13104-018-3239-3

**Published:** 2018-02-14

**Authors:** J. A. A. S. Jayaweera, F. Noordeen, S. Kothalaweala, F. N. N. Pitchai, M. L. M. Rayes

**Affiliations:** 1 Department of Microbiology, Faculty of Medical and Allied Sciences, Rajarata University of Sri Lanka, Anuradhapura, Sri Lanka; 2 Department of Microbiology, Faculty of Medicine, University of Peradeniya, Peradeniya, Sri Lanka; 3 Department of Pediatrics, Faculty of Medical and Allied Sciences, Rajarata University of Sri Lanka, Anuradhapura, Sri Lanka

**Keywords:** Human metapneumovirus, Acute respiratory tract infections, Children, Sri Lanka

## Abstract

**Objectives:**

The prevalence of hMPV infections in Sri Lanka has not been reported and here we report a case series of hMPV infection in children less than 5 years. Patients with ARTI were included from Teaching Hospital, Anuradhapura from March 2013 to August 2014. Indirect fluorescence assay was performed on nasopharyngeal aspirates for the identification of respiratory viruses [respiratory syncytial virus (RSV), parainfluenza virus 1, 2 and 3, influenza A and B and hMPV]. Moreover, reverse transcriptase-polymerase chain reaction was done to further confirm the hMPV infection.

**Results:**

In this case series, hMPV infection showed a range of respiratory symptoms from common cold to life threatening lower respiratory tract infections with varying severity. In some cases, the clinical presentation of hMPV infection was similar to the ARTI caused by RSV. hMPV co-infections with of RSV have also been seen in some cases of ARTI. A child delivered through cesarean section and birth order > 3 has an Odds ratio of 3.5 and 4.3 (95% CI) for developing co-infection with RSV compared to hMPV mono-infections. Lack of diagnostic facilities to identify the viral aetiology has contributed to the use of antibiotics indicating the need for establishing viral diagnostic facilities in the country.

**Electronic supplementary material:**

The online version of this article (10.1186/s13104-018-3239-3) contains supplementary material, which is available to authorized users.

## Introduction

Acute respiratory tract infection (ARTI) is one of the most common illnesses of childhood. ARTIs range from common cold, a mild self-limiting catarrhal syndrome to life threatening lower respiratory tract infection. Viruses account for most ARTIs and associated respiratory diseases [1, 2]. The most frequently reported viruses in newborns and children under 5 years with ARTI are respiratory syncytial virus (RSV), parainfluenza virus types 1, 2, 3, adenovirus, influenza virus types A, B, corona virus, Coxsackievirus, other enteroviruses, human boca virus and human metapneumovirus (hMPV) [1].

The hMPV, is the first member of a new genus *Metapneumoviru*s of the *Paramyxoviridae* family that infects humans [2, 3]. The RSV belongs to a separate genus within the *Paramyxoviridae* family [4]. hMPV was first isolated in 2001 in the Netherlands [2]. hMPV has been recently identified in nasopharyngeal aspirates (NPA) of children and adults with ARTI in various parts of the world [2, 5–8]. In temperate zones, hMPV infections peak in late winter and spring months but slightly later to the RSV peak in most studies [9]. We do not know the prevalence of hMPV in tropical countries and it might be due to lack of diagnostic facilities to detect respiratory viruses in these countries.

The clinical syndrome in the children infected with hMPV ranges from mild respiratory disease to severe bronchiolitis and pneumonia [10]. The children with severe disease require hospitalization [9]. Co-infection with RSV and hMPV causes severe disease compared to RSV or hMPV mono-infections [11, 12]. The risk factors for acquisition of viral ARTI is being studied in depth but the details of the viral co-infection have not being fully explored. Conversely, the prevalence of hMPV infection in Sri Lanka was not known and there are no published reports on the presence of hMPV in the country. Here we report a case series of hMPV infection in children less than 5 years of age in Sri Lanka.

## Main text

### Methods

**Fig. 1:**
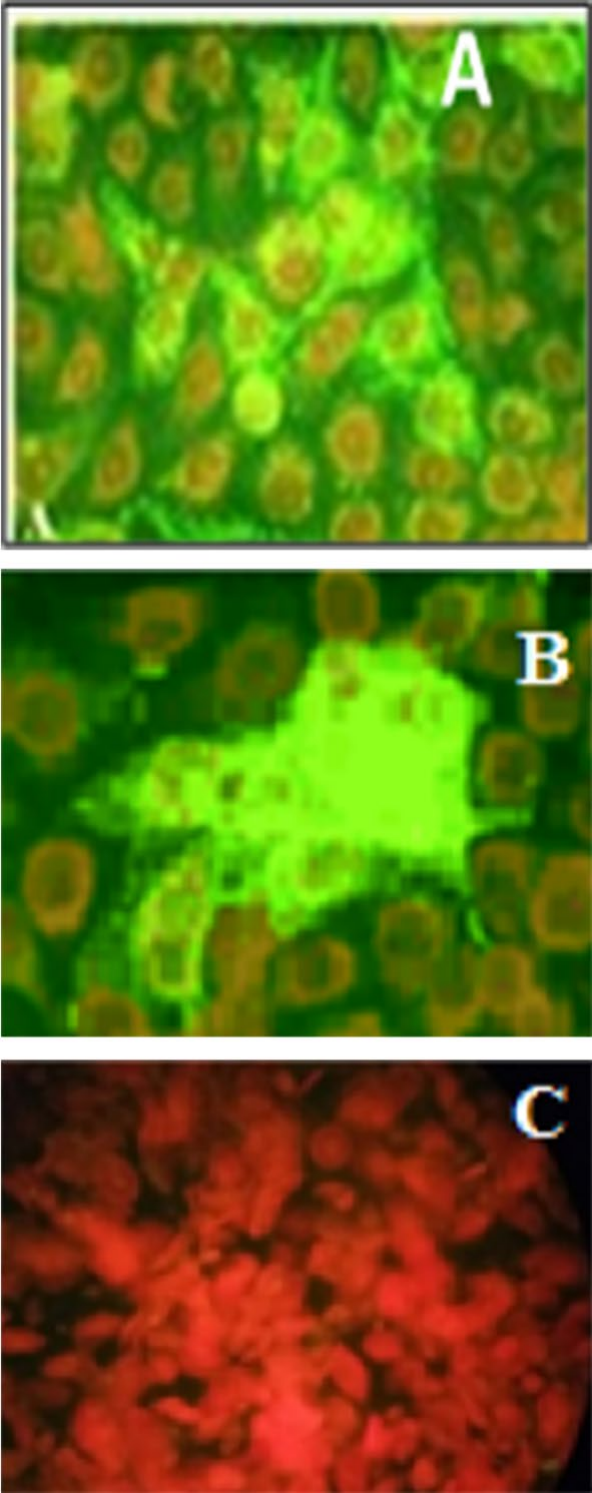
Immunofluorescence microscopic images of hMPV identified from NPAs of children. **A** hMPV, **B** positive control and **C** negative control (40 × 10× magnification)

The present study was conducted as a case series in which patients admitted with ARTI (age 1 to ≤ 60 months) were included from the Paediatric Professorial Unit, Teaching Hospital, Anuradhapura from March 2013 to August 2014. The informed consent was obtained from all parents or guardians prior to taking information and collecting nasopharyngeal aspirate (NPA) for viral diagnosis. Indirect immunofluorescence assay was performed using the DAKO IMAGEN™ UK (United Kingdom) as a screening test followed by a direct immunofluorescence assay (DFA) for specific identification of eight viruses (RSV, parainfluenza virus 1, 2 and 3, influenza A and B and hMPV) [12, 13]. Stained slides were examined under UV-epi fluorescence microscope (Leids, Germany) and intracellular nuclear and/or cytoplasmic granular apple green fluorescence emitting cells were considered as virus positive (Fig. 1). Test was employed under standard conditions while having simultaneous run of positive and negative controls provided by manufacturer. Reverse transcriptase-polymerase chain reaction (RT-PCR) of hMPV was done to further confirm the hMPV infection. A nested PCR was performed with L7 (5′-CACCCCAGTCTTTCTTGAAA-3′) from 11,471 to 11,490 positions in the detection of hMPV [14].

Data on clinical signs and symptoms, demography and risk factors for acquiring ARTI were collected using an investigator administered questionnaire by interviewing the parent or guardian (Additional file 1). Multivariable analyses were performed using a step wise logistic regression analysis to assess the risk factors for the development of RSV and RSV/hMPV co-infection. The risk factors included in the study were age at hospitalization, duration of the disease, gender of the child, ethnicity, weight for the given age (as a measure of acute malnutrition), height for the given age (as a measure of chronic malnutrition) and Hb%, gestational age to assess the maturity of the mother, mode of delivery and presence of concurrent medical conditions (congenital heart disease (CHD), chronic lung disease (CLD), asthma, cystic fibrosis, immunodeficiencies and epilepsy) and genetic disorders like Down’s syndrome-trisomy 21, neuromuscular disorders and pre-existing respiratory tract morbidity, parental passive smoking (father or any other family member regularly smoking cigarettes in the vicinity of the child), having house hold pets, presence of indoor (cooking using firewood) and outdoor air pollution (construction activities in the vicinity of the child), overcrowding (living area of the child is < 24 m^2^ and living > 2 people in that area [15]), day care attendance (child regularly attending to daycare), parent/guardian’s educational level (< grade 8, up to advanced level and graduates), experience of the caregiver (first child or having cared for > 1 child) and parent/guardian’s occupation. For the risk factor analysis, stepwise logistic regression and when appropriate chi-squired test was also used. Odds ratio was calculated and variables with a *p* value of < 0.05 and multivariate odds ratios with 95% confident interval (CI)s that did not include 1.0 were considered as significant. Statistical analysis was done using SAS software, Version 9.1 [16].

### Results

During the study period, 418 patients with ARTI were tested and 14 (0.03%) were diagnosed having hMPV infection. Six children had co-infection with RSV. hMPV infected patients were detected on two peaks, the first peak occurred in April 2013 and 2014 and the second peak occurred between December 2013 and January 2014.

Of the 14 hMPV infected children, nine were males (0.66%). Twelve children were from rural areas and 3 were from semi-urban areas. Mean age of the hMPV infected children was 18 months (6–36 months). Nine patients had fever as the presenting complaint; 4 children had only rales and 3 had only wheezing as the presenting complaint. One patient had watery diarrhoea but his stool culture was negative for bacterial pathogens causing diarrhoea.

Three cases were diagnosed having exacerbation of bronchiolitis following hMPV infection. One child had severe bronchiolitis associated with RSV/hMPV co-infection. One child had bilateral lower lobar pneumonia and severe bronchiolitis following hMPV infection; another child had right lower lobe pneumonia and severe bronchiolitis following RSV/hMPV co-infection. These children were admitted to intensive care unit and later discharged with follow-ups by paediatric clinic for the management of bronchiolitis. Two children had infective exacerbation of asthma with hMPV infection and the other had RSV/hMPV co-infection. Two patients had common cold following hMPV infection and one had common cold with RSV/hMPV co-infection. The disease spectrum and severity following hMPV and RSV/hMPV co-infection are described in Table 1.

**Table 1:** Disease spectrum and severity of ARTI following hMPV and RSV/hMPV co-infection

	hMPV (n = 9)	RSV/hMPV (n = 6)	Comments
Diseases			
Common cold	2	1	
Bronchiolitis	3	1	
Severe	2	1	
Moderate	0		
Mild	1		
Pneumonia			
Lobar	1	1	
Broncho	1	1	
Lobar pneumonia and severe bronchiolitis	1	1	Both were treated in ICU
IEA	1	1	

*IEA* infective exacerbation of asthma, *ICU* intensive care unit

Risk factor assessment for hMPV infection vs. RSV/hMPV co-infection showed that the children delivered through lower segmental cesarean section (LSCS) had an odds ratio of 3.5 (95% CI 3.5, 2.2–4.8) and birth order > 3 had an odds ratio of 4.3 (95% CI 4.3, 3.2–5.6) for developing RSV/hMPV co-infection compared to hMPV mono-infection. The duration of illness and the average hospital stay did not differ significantly in either hMPV or RSV/hMPV co-infection (Table 2).

**Table 2:** Risk factor assessment for the acquisition of hMPV and hMPV/RSV co-infection

	hMPV (n = 8)	hMPV/RSV co-infection (n = 6)	OR (95% CI)	p value
Duration of disease	6 ± 2.3 days	6 ± 2.2 days	–	0.2
Average hospital stays	5 ± 2 days	6 ± 2 days	–	0.3
Risk factors				
Malnutrition (Height-for-age *z*-score ≤ − 2)	2	2	1.1 (0.6–1.6)	0.3
Male sex	4	5	1.5 (0.9–1.9)	0.4
Low birth weight (< 2500 g)	2	2	1.1 (0.5–1.4)	0.4
Mode of delivery-LSCS	1	3	3.5 (2.2–4.8)	0.04*
Outdoor air pollution	3	2	1.2 (0.5–1.6)	0.6
Indoor air pollution	4	4	1.1 (0.6–1.5)	0.4
Passive smoking	3	4	1.3 (0.7–1.8)	0.4
Non-exclusive breastfeeding (during the first 4 months of life)	1	1	1.1 (0.5–1.4)	0.3
Lack of immunization (within the first 12 months of life)	0	0	0	0.0
Over crowding	4	4	1.1 (0.6–1.5)	0.3
Concomitant conditions				
Congenital heart diseases	1	1	1.1 (0.6–1.5)	0.3
Asthma	1	1	1.2 (0.7–1.8)	0.2
Known Immunodeficiency	1	–	0	–**
Epilepsy	1	–	0	–**
Mother’s experience as a caregiver	2	–	–	0.4
Mother’s education				
< grade 8	1	2	1.5 (0.6–1.9)	0.9
Day-care attendance	1	2	1.4 (0.7–1.8)	0.3
Trisomy 21	1	1	1.1 (0.7–1.4)	0.4
Birth order > 3	1	3	4.3 (3.2–5.6)	0.05*

*LSCS* lower segment caesarian section

* p < 0.05 was considered as significant

** Only one hMPV infected child and no children with RSV/hMPV co-infection were available for comparison

### Discussion

Viruses account for most of the respiratory tract infections in childhood [1, 12, 14]. Viral infections of the respiratory tract are often treated with antibiotics due to the absence of viral diagnostics to identify the viral aetiology. Thus, a proper diagnosis is crucial prior to initiating antibiotic treatment for bacterial ARTI or pneumonia [9, 15]. In developing countries, a lack of availability of diagnostic facilities contributes to the use of antibiotics and thus to development of antimicrobial resistance [9, 10]. The imaging studies and blood cell differential count may give a clue on the type of infective agent. However, in atypical pneumonias, getting an educated guess about the bacterial and viral causes are difficult. Hence, routine viral laboratory diagnosis is crucial and implementation of such facilities is highly warranted.

RSV is the most common respiratory viral pathogen causing hospitalization of thousands of children each year [2, 15]. Many of the affected children do not require hospitalization and some with severe respiratory disease are hospitalized or even managed in the intensive care unit (ICU). The children requiring ICU admission are typically young infants and those with co-morbidities. These children can be severely ill and require intubation and mechanical ventilation but most of the children recover and a very few succumb to the disease. Currently we are seeing the emergence of respiratory pathogens either due to change in antigenicity in influenza viruses or emergence and introduction of newly emerging viral pathogens like hMPV [12].

In this case series, hMPV infection showed a disease spectrum similar to that seen during RSV infection, common cold to life threatening pneumonia. Children delivered through LSCS appear to have less resistance to infection [11] and in our study also, children delivered through LSCS had a high risk of developing hMPV/RSV co-infection. A child with a birth order > 3 had a high risk of getting hMPV/RSV co-infection and this might be due to lack of care to subsequent children in bigger families. In a few cases, even without co- morbidities, children experienced severe hMPV infection needing ICU care. In many cases, RSV/hMPV co-infection resulted in similar disease spectrum to that of RSV infection.

Specific aetiological diagnosis of childhood ARTI is not performed routinely in Sri Lanka. But if it is done routinely it will invariably guide the clinicians on the use of antibiotics including antivirals. This case series indicates the importance of establishing laboratory diagnosis for viral ARTI. Furthermore, hMPV is a potential pathogen that needs to be tested in children with ARTI. A detailed epidemiological study is in progress to elucidate the prevalence and seasonality of childhood ARTI caused by a wider range of respiratory viruses including hMPV in Sri Lanka.

## Limitation/s

In this study, we have focused on inward patients only. Therefore, we may have missed a significant number of outpatients, who would have had milder form of hMPV associated ARTI.

## Additional file

**Additional file 1.** Questionnaire of the respiratory study. Questionnaire was used to gather demography and clinical manifestations in study subjects.

## Abbreviations

ARTI: acute respiratory tract infection
RSV: respiratory syncytial virus
hMPV: human metapeumovirus
NPA: nasopharyngeal aspirates
UK: United Kingdom
RT-PCR: reverse transcriptase-polymerase chain reaction
CHD: congenital heart disease
CLD: chronic lung disease
ICU: intensive care unit
