# Supplementary material for: A case series on common cold to severe bronchiolitis and pneumonia in children following human metapneumovirus infection in Sri Lanka
Source: BMC Res Notes. 2018 Feb 14;11:127. doi: 10.1186/s13104-018-3239-3 (PMC5813322; doi:10.1186/s13104-018-3239-3)
Supplement: Supplementary file 1 — Additional file 1. Questionnaire of the respiratory study. Questionnaire was used to gather demography and clinical manifestations in study subjects. [file 13104_2018_3239_MOESM1_ESM.docx]

**Questionnaire for investigating the**

**“Viral burden in acute respiratory tract infections in children in selected areas of Sri Lanka”**

**Date: …………………………………….**

**Sample No: …………/Gampola/Anuradhapura**

**BHT: ………………...................................**

**General Information**

1. Name…………………………………………………………………………………………………………………….
2. Age …………… (yrs)/…………………..(months)
3. Sex Male F Female

1. Weight…………………Kg Height/Length……………..cm
2. Weight to height………………………. Wt/age……………Percentile in CHDR Ht/age……………..percentile
3. Gomez-………………………………………
4. Age <1 Breast feeding Yes No

1. Number of siblings …………….. Day care Yes No
2. Urban Semi urban Rural Estate
3. Ethnicity Sinhala Tamil Muslim Other

**Brief History**

1. Birth weight …………………………………Kg

1. Maturity in……………Weeks 2.1 Mode of delivery NVD/LSCS if LSCS done

2.2 LSCS indication……………………………………………

1. Presenting Complaint ……………………………………………………Duration………………….../Days

4. Had similar condition(s) …………….in last 3 months

Previous Diagnosis……………………………………………………………………………………………...........

5. Congenital anomalies

Cardiac Yes No f if yes ……………………………………………………………..

Respiratory Yes No if yes ……………………………………………………………..

6. Immunodeficiency Yes No if yes ……………………………………………………………..

On long term drugs (>2/52 regular) ……………………………………………………………………………….

7. Past medical history

Cystic fibrosis Yes No …………………………………………………

Bronchial asthma yes No **If yes**

infective exacerbation yes No

8. Parent/grandparents/any Smoking at home Yes No

Domestic industries………………………………………………………………………………………………………………

**Nearby –if Yes**

Metal crusher harvesting machines

Dolomite factory Rubber industry

Carpentry Garage

Any other……………………………………………………………………………………………………………………………….

9. Parent/s’ occupation………………………………………………………………………………………………………….

10. Monthly income……………………………………………………………………………………………………………….

**Symptom Analysis Physical Examination**

| **Signs/symptoms** | **Yes** | **No** |
| --- | --- | --- |
| Fever |  |  |
| Cough |  |  |
| Runny nose |  |  |
| Difficulty in breathing |  |  |
| Headache-Frontal |  |  |

| Respiratory rate | /min |
| --- | --- |
| Grunting |  |
| Chest wall recession |  |
| Cyanosis |  |

- Body temperature ……………^o^C
- Associated with Conjunctivitis

Arthritis

Diarrhoea

Any other symptoms……………………………………………………………………………………………………………

Chest X-ray findings……………………………………………………………………………………………………………..

……………………………………………………………………………………………………………….

Full Blood Count Hb%.....................g/dL

WBC………………..

Neutrophiles…………………% Lymphocytes ………………..%

Eosinophiles …………………%

C reactive protein………………………………

Saturation (SPO_2_)…………………..% (on air)

Oxygen therapy given Yes No

**Diagnosis…………………………………………………………………………………………………………………………..**

***Intensive Care Unit Admissions*** Yes No Duration………………………..

***High Dependency Unit Admissions*** Yes No Duration………………………..

Duration of hospital stay 1-2 days

3-5 days

>7 days

Antibiotic given Yes No if prescribed

Antibiotic and the Dose………………………………………………………………………………………………….

Other treatments IV fluids

……………………………………………………………….. ………………………………………………………

………………………………………………………………… ………………………………………………………

…………………………………………………………………. ………………………………………………………

…………………………………………………………………

…………………………………………………………………..

…………………………………………………………………..

…………………………………………………………………..
